# Thioredoxin-1 Ameliorates Oxygen-Induced Retinopathy in Newborn Mice through Modulation of Proinflammatory and Angiogenic Factors

**DOI:** 10.3390/antiox11050899

**Published:** 2022-04-30

**Authors:** Junichi Ozawa, Kosuke Tanaka, Yukio Arai, Mitsuhiro Haga, Naoyuki Miyahara, Ai Miyamoto, Eri Nishimura, Fumihiko Namba

**Affiliations:** Department of Pediatrics, Saitama Medical Center, Saitama Medical University, Kawagoe 3508550, Japan; ozajun321@gmail.com (J.O.); kotanaka0403@gmail.com (K.T.); kumatarou13@yahoo.co.jp (Y.A.); mitsuhiro.haga@indigo.plala.or.jp (M.H.); ma03084ma@yahoo.co.jp (N.M.); ma_tu_2005xp@yahoo.co.jp (A.M.); erimura1120@gmail.com (E.N.)

**Keywords:** oxygen-induced retinopathy, retinopathy of prematurity, retinal blood vessel, thioredoxin-1, newborn mouse, hyperoxia, angiogenic factor

## Abstract

Oxygen-induced retinopathy (OIR) is an animal model for retinopathy of prematurity, which is a leading cause of blindness in children. Thioredoxin-1 (TRX) is a small redox protein that has cytoprotective and anti-inflammatory properties in response to oxidative stress. The purpose of this study was to determine the effect of TRX on OIR in newborn mice. From postnatal day 7, C57BL/6 wild type (WT) and TRX transgenic (TRX-Tg) mice were exposed to either 21% or 75% oxygen for 5 days. Avascular and neovascular regions of the retinas were investigated using fluorescence immunostaining. Fluorescein isothiocyanate-dextran and Hoechst staining were used to measure retinal vascular leakage. mRNA expression levels of proinflammatory and angiogenic factors were analyzed using quantitative polymerase chain reaction. Retinal histological changes were detected using immunohistochemistry. In room air, the WT mice developed well-organized retinas. In contrast, exposing WT newborn mice to hyperoxia hampered retinal development, increasing the retinal avascular and neovascular areas. After hyperoxia exposure, TRX-Tg mice had enhanced retinal avascularization compared with WT mice. TRX-Tg mice had lower retinal neovascularization and retinal permeability during recovery from hyperoxia compared with WT mice. In the early stages after hyperoxia exposure, VEGF-A and CXCL-2 expression levels decreased, while IL-6 expression levels increased in WT newborn mice. Conversely, no differences in gene expressions were observed in the TRX-Tg mouse retina. IGF-1 and Angpt1 levels did not decrease during recovery from hyperoxia in TRX-Tg newborn mice. As a result, overexpression of TRX improves OIR in newborn mice by modulating proinflammatory and angiogenic factors.

## 1. Introduction

Retinopathy of prematurity (ROP) is one of the most common causes of childhood blindness, mainly affecting extremely premature infants [1]. Many premature infants require supplemental oxygen for survival after birth. This sudden increase in oxygen supply following a hypoxic environment in utero or increased atmospheric oxygen causes abnormal vascular development. During ROP development, abnormal retinal blood vessels form sprouts and branches extending across the retinal surface and into the vitreous, resulting in pathological retinal neovascularization and intravitreal neovascularization [2]. Currently, ROP treatments include laser photocoagulation, cryotherapy, and anti-vascular endothelial growth factor (VEGF) therapy that has limited efficiency. Hopefully, improved treatment and prevention methods will be developed in the future [3]. Several studies have been conducted on the effects of antioxidants on ROP, such as vitamin E and glutathione peroxidase-1. These studies concluded that antioxidant capacity plays an important role in preventing and lowering the risk of severe ROP in preterm retinas [4,5,6,7].

Thioredoxin-1 (TRX) is an endogenous antioxidant enzyme that contains a redox-active disulfide/dithiol within the conserved Cys–Gly–Pro–Cys sequence [8]. TRX protects cells from oxidative stress by scavenging reactive oxygen species (ROS) [8,9] while simultaneously modulating chemotaxis and inhibiting leukocytic infiltration to the inflammation sites [10]. Previous research demonstrated that TRX transgenic (TRX-Tg) mice or mice in which recombinant human TRX is systemically administered are resistant to injury in different animal models of human diseases, including viral pneumonia [11], acute lung injury [12,13], pancreatitis [14,15], myocarditis [16], chronic obstructive pulmonary disease [17], indomethacin-induced gastric injury [18], lipopolysaccharide-induced preterm delivery [19], hyperoxic lung injury [20], and brain ischemia [21]. However, no research reported on the assessment of the effect of TRX on the ROP animal model.

The mouse model of oxygen-induced retinopathy (OIR) has been commonly used in in vivo studies on ROP [22,23]. This is due to fact that the OIR model mouse adequately reproduces the vascular regression and neovascularization phases of ROP and can be used in preclinical experiments to evaluate potential therapeutic options. Hyperoxia is responsible for ROS production by increasing superoxide due to the absence of a retina bloodstream autoregulation system [24]. Hypoxia can also activate nitric oxide synthase and nicotinamide adenine dinucleotide phosphate oxidase, both of which generate ROS and are involved in OIR [25]. Using this OIR model mouse, we examined the effect of TRX in the OIR mouse model with the ultimate goal of determining the effectiveness of overexpression of recombinant human TRX as a novel therapeutic agent for the prevention and treatment of ROP in premature infants.

## 2. Materials and Methods

### 2.1. Animals

**Figure 1:**
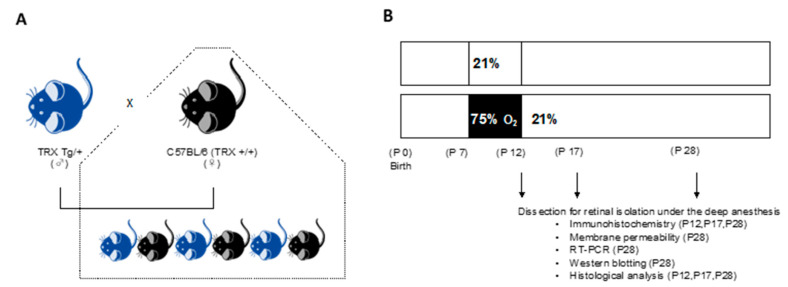
Experimental design. (**A**) The breeding scheme and genotypes of the mice that were studied. Heterozygous TRX-Tg male and WT female mice were mated to produce either TRX heterozygous or WT littermates. (**B**) Experimental method of exposing newborn mice to hyperoxia. P7 pups were randomly assigned to normal room air (21% O_2_) or hyperoxic (75% O_2_) conditions with a nursing mother. On P12, some mice were released to room air and were able to recover until P12, P17, and P28. TRX, thioredoxin-1; Tg, transgenic; WT, wild type; P, postnatal day.

All protocols and procedures were approved by the Animal Care and Use Committee of Saitama Medical University (permit no.: 2256, 2303, 2552, 2809, 3099). TRX-Tg mice were firstly developed by Takagi et al. [26]. Briefly, human TRX cDNA was inserted between the β-actin promoter and terminator. The transgene was extracted from the plasmid using VspI and XbaI digestion, purified, and used to create transgenic mice. The transgene was microinjected into the pronucleus of the fertilized eggs from C57BL/6 mice. TRX-Tg mice were repeatedly backcrossed with C57 BL/6 mice for at least 12 generations and maintained in the animal facility of Saitama Medical Center, Saitama Medical University. Heterozygous TRX-Tg male and wild type (WT) female mice were mated to produce either TRX heterozygous or WT littermates (Figure 1A). After mating overnight, vaginal plugs are tested, and the day of plug development is termed embryonic day 0. The mice were given a 12-h light/12-h dark schedule and had free access to tap water and a standard laboratory diet. Genotyping was conducted on all mice. The mouse’s tail was excised approximately 5 mm and soaked in cell lysis solution (Thermo Fisher Scientific, Waltham, MA, USA) and protein kinase K solution (Thermo Fisher Scientific, Waltham, MA, USA) overnight at 55 °C. Then, following the manufacturer’s recommendations, DNA was extracted using a protein precipitation solution (Thermo Fisher Scientific, Waltham, MA, USA) and DNA hydration solution (QIAGEN, Hilden, Germany). Gotaq (Promega KK, Madison, WI, USA) and oligonucleotide primers (forward primer, 5′-CCGCTCGTCAGACTCCAGC-3′; and reverse primer, 5′-CTGGTTATATTTTCAGAAAACATG-3′) (Eurofins Genomics, Ebersberg, Germany) were added to the isolated DNA, and polymerase chain reaction (PCR) was conducted to determine the genotype. In conducting ocular animal tests, we followed the Statement for the Use of Animals in Ophthalmic and Vision Research. The number of animals used in each experimental group was specified in each Figure.

### 2.2. Neonatal Hyperoxic Exposure and Recovery

The day of birth is defined as postnatal day (P) 0, all newborn pups were bred in room air. From P7 to P12, the OIR mouse was developed by exposing it to 75% oxygen. P7 pups were placed in a 75% oxygen chamber and bred with a nursing mother. To avoid oxygen toxicity in the dams and to eliminate maternal effects between groups, the nursing mother was switched between hyperoxia and normal room air every 24 h. P7 pups were given either normal room air (21% O_2_) or hyperoxic (75% O_2_) conditions. In the animal chamber, the levels of O_2_ and CO_2_ were continuously monitored and adjusted (BioSpherix, Redfield, NY, USA). On P12, hyperoxia-exposed mice were returned to room air and recovered until P12, P17, and P28 for further analysis (Figure 1B). Both eyes of neonatal mice were removed after they were profoundly anesthetized. According to prior studies, the end of oxygen exposure (P12) is the vascular regression phase. The P17 is the most active neovascularization phase, and the completed phase of neovascularization was also assessed at P28 [27,28].

### 2.3. Immunohistochemistry of Retinal Vessels

Pentobarbital (50 mg/kg) was administered intraperitoneally to mice, and both eyes were enucleated under deep anesthesia. The enucleated right eye was fixed in 4% paraformaldehyde (PFA) for 30–40 min at 4 °C. The cornea, lens, uvea, and sclera were removed under the stereomicroscope (SZ61, Olympus, Tokyo, Japan) after the fixed eye was immersed in phosphate-buffered saline (PBS). The retina was fixed in 4% PFA overnight and stored in methanol at −20 °C.

Retinal whole-mount staining was conducted according to methods reported previously [28]. The retina was incubated overnight with a rat monoclonal anti-CD31 (PECAM-1) antibody (1:500, clone MEC 13.3, BD Biosciences, San Diego, CA, USA), followed by a Cy3-conjugated goat polyclonal anti-rat IgG antibody (1:400, AB_2338240, Jackson ImmunoResearch, West Grove, PA, USA) after being blocked in a blocking solution (Protein Block Serum-Free Ready-to-Use, Dako, Santa Clara, CA, USA) for 0.5 h at room air temperature. Next, the retina was washed with PBS containing 0.5% Tween 20 (Sigma-Aldrich, St. Louis, MO, USA), mounted in VECTASHIELD (Vector Laboratories, Burlingame, CA, USA), observed under a fluorescence microscope (BZ-X800, Keyence, Osaka, Japan), and assessed with ImageJ software version 1.51 (NIH, Bethesda, MD, USA) for the measurements of the avascular area, neovascular area, and number and diameter of retinal arteries and veins. All evaluation procedures were conducted in a masked fashion by a single observer (J.O.). We differentiate endothelial cells stained with anti-CD31 from the background by applying threshold fluorescence intensity values. The avascular network ratio was computed by dividing the avascular area by the entire retinal area using the number of pixels, and the neovascular area ratio was derived by dividing the neovascular area by the total retinal area by 100 (%) [29]. The numbers and diameters of the arteries and veins were measured in an area that is 500 μm away from the optic disc. Five to six retinas were examined in each group.

### 2.4. Retinal Vascular Permeability

The permeability of the mouse’s retinal vascular system was tested on P28 using Nakamura’s approach [30]. To achieve profound anesthesia, mice were given a 50 mg/kg intraperitoneal injection of pentobarbital. The chest chamber of the mouse was then accessed. Each mouse was perfused with 1 mL of PBS containing 100 mg/mL of bisbenzimide Hoechst 33342 (molecular weight, 561.93; Sigma-Aldrich, St. Louis, MO, USA) and 20 mg/mL of fluorescein isothiocyanate-dextran (FITC-dextran) (molecular weight, 2,000,000; Sigma-Aldrich, St. Louis, MO, USA) thorough a needle inserted into the left ventricle. A single observer (J.O.) masked the individual retinas, viewed them under a fluorescence microscope, and analyzed them using ImageJ software. The stained areas of Hoecht33342 and FITC-dextran were measured in pixels at a distance of 500 μm from the optic disc. We used a method reported previously to calculate the membrane permeability rate [30]. From each group of four to six retinas, three to four portions of each specimen were selected.

Permeability rate = [(Hoechst 33342 area) − (area of FITC-dextran and Hoechst 33342 colocalization)])/(FITC-dextran area).

### 2.5. Western Blot Analysis of the Retina

The amounts of occludin and claudin-5 were measured using western blots. The retinas of enucleated eyes were removed and snap-frozen in liquid nitrogen. Using an ultrasonic homogenizer (SONICSTAR 85 AS ONE, Osaka, Japan), the tissue was homogenized in T-PER buffer (Thermo Fisher Scientific, Waltham, MA, USA) with a protease inhibitor (Thermo Fisher Scientific, Waltham, MA, USA). The lysate was centrifugated for 10 min at 4 °C at 12,000× *g*, and the supernatant was used for the experiment. An equal amount of protein (70 μg) was mixed with sample buffer and 10% 2-mercaptoethanol and subjected to sodium dodecyl sulfate-polyacrylamide gel electrophoresis. A polyvinylidene difluoride membrane was used to blot the isolated protein. After that, the membranes were treated with anti-occludin mouse monoclonal antibody (Santa Cruz Biotechnology, Santa cruz, CA, USA) (1:200), anti-claudin-5 mouse monoclonal antibody (Santa Cruz Biotechnology, Santa cruz, CA, USA) (1:500), or anti-β-actin polyclonal rabbit antibody (GeneTex, Irvine, CA, USA) (1:5000), followed by HRP-labeled secondary antibodies (Santa Cruz Biotechnology, CA, USA). Chemiluminescence was detected using a digital imaging system (Bio-Rad ChemiDoc XRS+; Bio-Rad Laboratories, Hercules, CA, USA) and the Amersham ECL Prime Western blot detection reagent (GE Healthcare, Little Chalfont, UK) using. The collected images were examined using Image Lab Software (Bio-Rad ChemiDoc XRS+; Bio-Rad Laboratories, Hercules, CA, USA). Six retinas were examined in each group.

### 2.6. RNA Extraction and Quantitative Real-Time PCR Analysis in the Retina

Quantitative real-time PCR was used to determine the levels of retinal mRNA gene expression. Five to six retinal tissue samples were used in each group. The isolated retina was submerged in RNAlater (Thermo Fisher Scientific, Waltham, MA, USA) at room temperature after the left eye was enucleated and stored at −20 °C until further studies. The tissue was homogenized using BioMasher II (Nippi, Tokyo, Japan). The RNA was isolated with TRIzol Reagent (Thermo Fisher Scientific, Waltham, MA, USA) and reverse-transcribed with the High-Capacity cDNA Reverse Transcription Kit (Applied Biosystems, Foster City, CA, USA). cDNA was amplified through PCR using TaqMan probes (Applied Biosystems, Foster City, CA, USA) for vascular endothelial growth factor A (Vegf-a, Mm00437306_m1), insulin-like growth factor -1 (Igf1, Mm00439560_m1), angiopoietin-1 (Angpt1, Mm00456503_m1), interleukin-6 (Il-6, Mm00446190_m1), heme oxygenase-1 (Ho-1, Mm00516005_m1), interleukin-1β (Il-1β, Mm00434228_m1), and chemokine (C-X-C motif) ligand 2 (Cxcl2, Mm00436450_m1) with the TaqMan Universal PCR Master Mix (Applied Biosystems, Foster City, CA, USA). The 7500 Fast Real-Time PCR System (Applied Biosystems, Foster City, CA, USA) was used to perform all PCR experiments. Each sample was normalized toβ-glucuronidase (Mm01197698_m1) (Applied Biosystems, Foster City, CA, USA), and relative mRNA expression levels were determined using the comparative critical threshold method.

### 2.7. Retinal Membrane Histological Analysis

Pentobarbital (50 mg/kg) was injected intraperitoneally to sacrifice the mice. Both eyeballs were enucleated and fixed for 24 h at 4 °C with 4% PFA. They were rinsed in PBS after fixation and stored in 70% ethanol at 4 °C. Paraffin-embedded retinal tissue was placed onto glass slides in 3-µm-thick sections. The light microscope (BX50, Olympus, Tokyo, Japan) was used to examine paraffin-embedded retinal tissue sections stained with hematoxylin and eosin. Each retinal section was separated into two parts, the central and peripheral regions, based on the length of the optic disc (central, 50 µm; peripheral, 500 µm). The thicknesses of the inner plexiform layer (IPL), inner nuclear layer (INL), outer plexiform layer (OPL), and outer nuclear layer (ONL), as well as the number of cells in the ganglion cell layer (GCL), were measured. On P12, P17, and P28, five retinal tissues were observed in each group.

### 2.8. Statistical Analysis

Data were examined using a two-way analysis of variance followed by the Tukey–Kramer test. EZR software (Saitama Medical Center, Jichi Medical University, Saitama, Japan) was used in the statistical analysis. A *p*-value < 0.05 was considered statistically significant. All data are presented as mean ± SEM.

## 3. Results

### 3.1. Immunohistochemistry of Retinal Vessels

#### 3.1.1. Retinal Avascular Area

**Figure 2:**
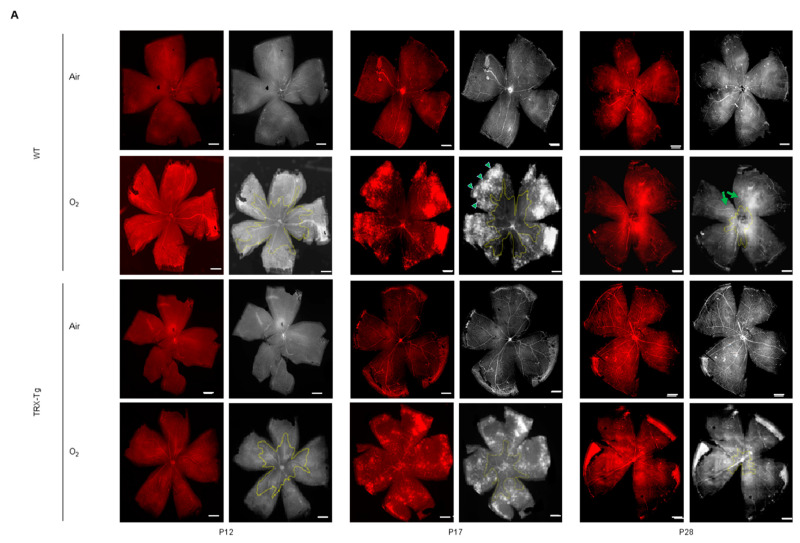

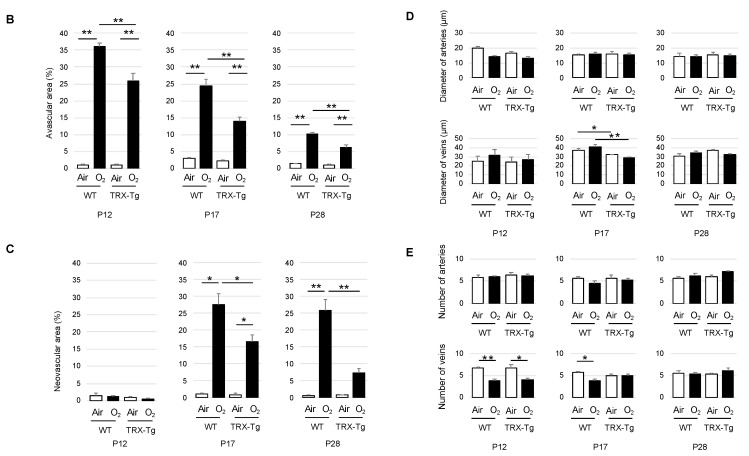
Immunohistochemical staining of mouse retinal vessels with anti-CD31 antibody. (**A**) In each group, the representative original image (left column) and the processed image for analysis (right column). An avascular field is indicated by the yellow line. The green arrowheads indicate the neovascular area. Green arrows show tortuous blood vessels. Each scale bar is 250 μm. (**B**) around the optic disc, there is an avascular retinal region. (**C**) The neovascular zone. (**D**) The number of retinal arteries. (**E**) The number of retinal veins. * *p* < 0.05, ** *p* < 0.01, P12, *n* = 5; P17, *n* = 6; P28, *n* = 5; P, postnatal day; WT, wild type; TRX-Tg, thioredoxin-1 transgenic.

We assessed P12, P17, and P28 avascular retinal areas. Regardless of genotype, the retinal avascular area was significantly larger in mice subjected to neonatal hyperoxia compared with mice exposed to room air (Figure 2A). The avascular area of the TRX-Tg retina exposed to hyperoxia was significantly less than that of the WT retina exposed to hyperoxia. This tendency was the same on any postnatal day (P12, 25.8% (±5.21) vs. 36.0% (±2.47), *p* < 0.01; P17, 13.8% (±2.83) vs. 24.4% (±4.97), *p* < 0.01; P28, 6.02% (±2.08) vs. 10.1% (±1.50), *p* < 0.01) (Figure 2B).

#### 3.1.2. Retinal Neovascular Area

No retinal neovascular region is visible on P12 in any of the groups. From P17, after returning to room air from hyperoxic conditions, retinal neovascular development began to occur. The retinal neovascular area was small in room air at P17 and P28, and there was no significant difference between the WT and TRX-Tg groups. After recovery from hyperoxia exposure, both WT and TRX-Tg started to form neovascular areas. The neovascular area was significantly bigger in the hyperoxia-exposed retina than in the retina exposed to room air in both genotypes. Among the retina exposed to hyperoxia, the TRX-Tg displayed a smaller neovascular area than the WT (P17, 16.5% (±4.95) vs. 27.6% (±7.58), *p* < 0.05; P28, 7.32% (±2.74) vs. 25.8% (±7.45), *p* < 0.05) (Figure 2C).

#### 3.1.3. Morphological Difference in Retinal Arteries and Veins

We found many tortuous retinal vessels in WT mice subjected to hyperoxia, but just a few in TRX-Tg mice exposed to hyperoxia. At P12, P17, and P28, there were no significant differences in retinal artery diameter between any of the groups. The retinal vein width of WT and TRX-Tg mice with and without hyperoxia exposure was not significantly different at P12. On P17, the diameter of veins in the TRX-Tg retina was significantly smaller than WT under both room air and hyperoxic circumstances (room air, 32.0 (±0.41) vs. 37.1 (±1.85) μm, *p* < 0.05; hyperoxia, 28.7 (±1.05) vs. 40.7 (±3.03) μm, *p* < 0.01). On the other hand, the P28 retina did not display any difference in the diameter of veins between each group (Figure 2D).

The number of arteries did not significantly differ among all groups on P12, P17, and P28. In both WT and TRX-Tg mice on P12, the number of veins in the retina subjected to hyperoxia was lower than that in the retina exposed to room air (WT, 3.83 (±0.75) vs. 6.60 (±0.55), *p* < 0.01; TRX-Tg, 4.00 (±0.71) vs. 6.62 (±1.82), *p* < 0.05). On P17, the number of veins was significantly smaller in the retina subjected to hyperoxia than those under normoxic conditions in WT mice (3.8 (±0.37) vs. 5.67 (±0.33), *p* < 0.05). However, in TRX-Tg, the significant difference between the retina subjected to hyperoxia and the retina exposed to room air vanished (5.0 (±0.41) vs. 5.0 (±0.32), *p* > 0.05). Furthermore, the P28 retina did not show any difference in the number of veins in each group (Figure 2E).

### 3.2. Retinal Vascular Permeability

**Figure 3:**
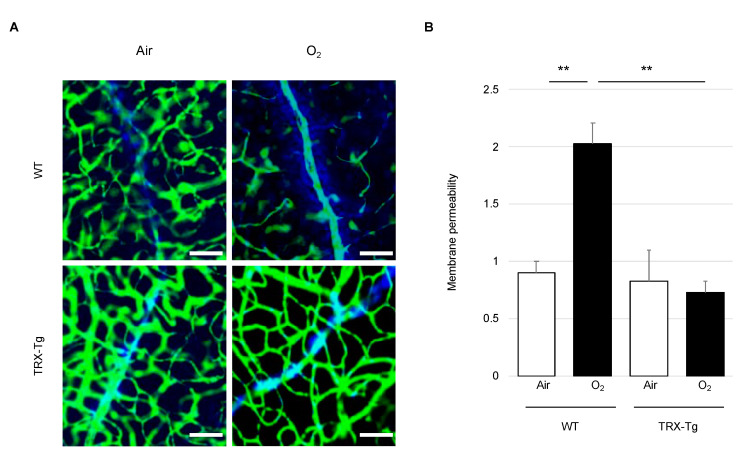
Mouse retinal vascular permeability. (**A**) Original picture immunostaining for blood vessel permeability on P28. The green network depicts FITC-dextran stained blood vessels, whereas the blue network reveals Hoechst 3324-stained cells. The scale bar is 20 μm. (**B**) Blood vessel permeability is calculated by [(Hoechst 33342 area) − (FITC-dextran + area around the blood vessel stained with Hoechst 33342)]/(FITC-dextran area) in each group. ** *p* < 0.01. Results are displayed as mean ± standard error. WT room air, *n* = 4; WT hyperoxia, *n* = 6; TRX-Tg room air, *n* = 6; TRX-Tg hyperoxia, *n* = 6. WT, wild type; TRX-Tg, thioredoxin-1 transgenic.

Extravasated Hoechst 33342 from vessels labeled the nucleus of surrounding cells, while injected FITC-dextran dye marked the vascular lumen (Figure 3A). In the WT mouse retina exposed to hyperoxia, more Hoechst 33342 flowed out of blood vessels compared to those in WT under room air circumstances on P28 (2.02 (±0.37) vs. 0.90 (±0.20), *p* < 0.01), showing that the retinal permeability rate was significantly higher in the hyperoxia-exposed retina in WT mice. Among hyperoxia-exposed mice, the permeability rate in TRX-Tg mice was significantly lower than that in WT mice (0.72 (±0.21) vs. 2.02 (±0.37), *p* < 0.01) (Figure 3B).

### 3.3. Expression Levels of Tight Junction Proteins

**Figure 4:**
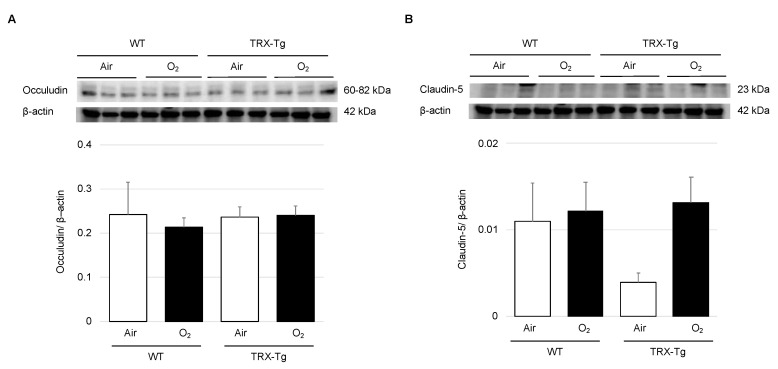
Expression levels of tight junction protein in the mouse retina. (**A**) Occludin (60–82 kDa) was tested. β-actin (42 kDa) was used as a control. (**B**) Claudin-5 (23 kDa) was examined. β-actin was used as a control. The results are displayed as mean ± standard error. Six retinas from each group were used (*n* = 6). WT, wild type; TRX-Tg, thioredoxin-1 transgenic.

There were no significant differences in the protein expression levels of claudin-5 and occludin in any group (Figure 4).

### 3.4. Retinal mRNA Expression Levels of Proinflammatory and Angiogenic Factors

Because TRX had antioxidant and chemotaxis-modulating functions and reduced leukocytic infiltration to inflammation sites [17,20], the retinal mRNA expression levels of proinflammatory cytokines, chemokines, and antioxidants, including Il-6, Il-1β, and HO-1, were obtained using quantitative real-time PCR. Angiogenic factors, such as VEGF-A, IGF-1, CXCL2, and Angpt1, were also tested because neovascularization is an essential etiology of ROP.

**Figure 5:**
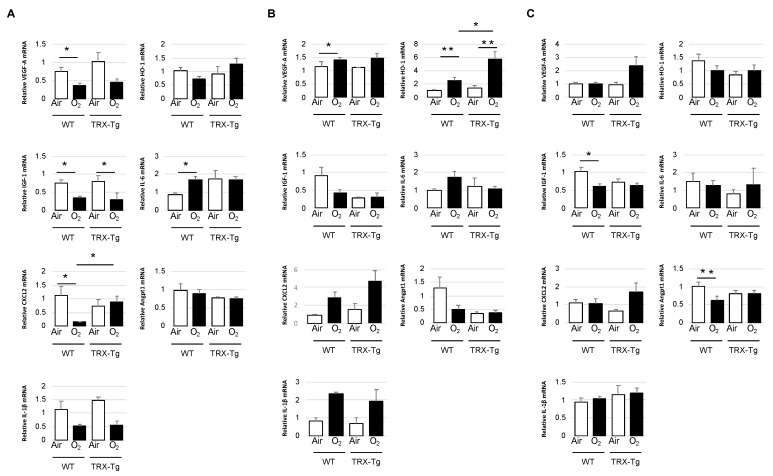
Mouse retinal mRNA expression levels of VEGF-A, IGF-1, CXCL2, IL-1β, HO-1, IL-6, and Angpt1. Quantitative real-time PCR was carried out at (**A**) P12, (**B**) P17, and (**C**) P28. * *p* < 0.05, ** *p* < 0.01. Results are displayed as mean ± standard error (*n* = 5–6). P, postnatal day; WT, wild type; TRX-Tg, thioredoxin-1 transgenic; VEGF-A, vascular endothelial growth factor-a; IGF-1, insulin-like growth factor-1; CXCL-2, chemokine ligand 2; IL-1β, interleukin-1β; HO-1, heme oxygenase-1; Il-6, interleukin-6; Angpt1, angiopoietin- 1.

On P12, in the WT retina exposed to hyperoxia, VEGF-A and CXCL2 expression levels were significantly lower than those in WT under room air conditions (0.37 (±0.13) vs. 0.74 (±0.25), *p* < 0.05; 0.15 (±0.02) vs. 1.03 (±0.74), *p* < 0.05). On the other hand, there was no significant difference in their retinal expression levels between TRX-Tg mice exposed to room air and hyperoxia (1.03 (±0.64) vs. 0.45 (±0.21), *p* = 0.06; 0.73 (±0.55) vs. 0.89 (±0.47), *p* = 0.32). Moreover, the hyperoxia-exposed TRX-Tg retina had significantly higher mRNA expression levels of CXCL2 compared to the hyperoxia-exposed WT retina (0.89 (±0.47) vs. 0.15 (±0.02), *p* < 0.05). Although there was no difference in IL-6 expression levels between TRX-Tg retinas exposed to hyperoxia and room air (1.72 (±0.40) vs. 1.76 (±1.08), *p* = 0.94), retinal IL-6 expression levels in WT mice exposed to hyperoxia were significantly higher than those in mice exposed to room air (1.72 (±0.33) vs. 0.86 (±0.17), *p* < 0.01). Retinal IGF-1 expression levels were significantly decreased after hyperoxic exposure in both WT and TRX-Tg mice (0.34 (±0.11) vs. 0.75 (±0.20), *p* < 0.01; 0.30 (±0.06) vs. 0.80 (±0.43), *p* < 0.05). Other mRNAs, such as IL-1β, HO-1, and Angpt1, did not reveal any difference in each group at P12 (Figure 5A).

After 5 days of recovery, at P17, retinal VEGF-A expression levels were significantly higher in WT mice exposed to hyperoxia compared to WT mice under room air conditions (1.41 (±0.22) vs. 1.01 (±0.15), *p* < 0.05). In contrast, no significant difference in retinal VEGF-A expression levels was seen between TRX-Tg mice subjected to hyperoxia and those exposed to room air (1.65 (±0.52) vs. 1.13 (±0.01), *p* = 0.145). Retinal HO-1 expression levels were significantly higher in both WT and TRX-Tg exposed to hyperoxia compared with those under room air conditions (2.50 (±1.63) vs. 1.01 (±0.39), *p* < 0.01; 5.76 (±3.77) vs. 1.15 (±1.01), *p* < 0.01). However, compared with the hyperoxia-exposed WT retina, HO-1 mRNA expression levels were significantly increased in the hyperoxia-exposed TRX-Tg retina (2.50 (±1.63) vs. 5.76 (±3.77), *p* < 0.05) (Figure 5B).

On P28, in the WT mouse retina exposed to hyperoxia, the mRNA expression levels of IGF-1 and Angpt-1 were reduced than those under room air conditions (0.61 (±0.15) vs. 1.03 (±0.32), *p* < 0.05; 0.63 (±0.24) vs. 1.13 (±0,11), *p* < 0.01). However, there were no significant changes in IGF-1 and Angpt-1 expression levels between the TRX-Tg retina subjected to hyperoxia and room air (0.63 (±0.18) vs. 0.74 (±0.19), *p* = 0.37; 0.81 (±0.24) vs. 0.81 (±0.22), *p* = 0.98) (Figure 5C).

### 3.5. Retinal Membrane Histology

**Figure 6:**
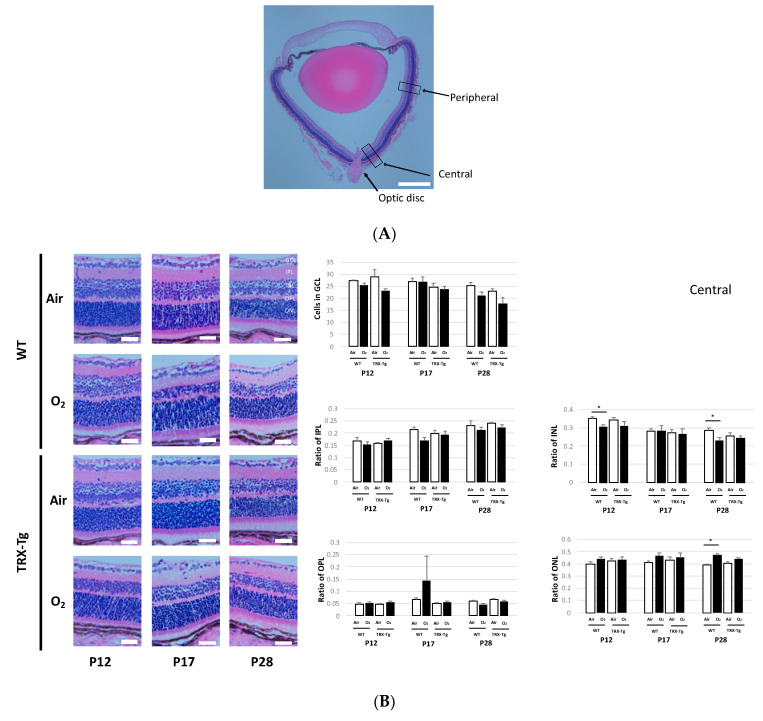

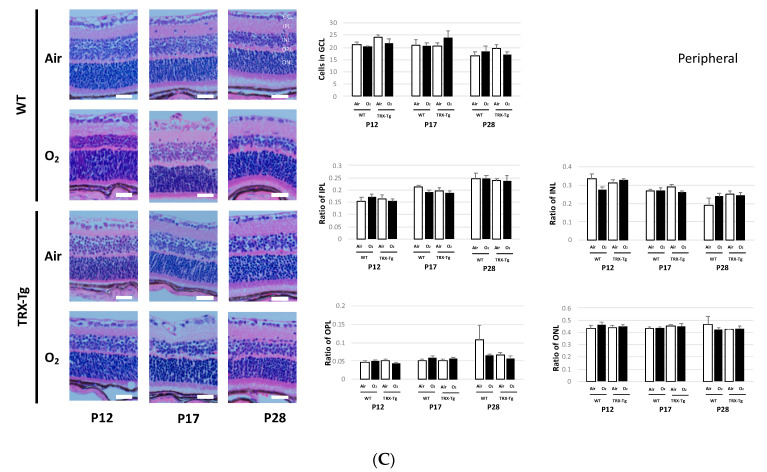
(**A**) Histology of the retinal membrane was performed at this location. The central part, 50 μm away from the optic disc; the peripheral part, 500 μm from the optic disc. The scale bar is 200 μm. (**B**) Central and (**C**) peripheral parts of retinal histology. The retina was stained with hematoxylin and eosin. The scale bar is 20 μm. * *p* < 0.05. GCL, ganglion cell layer; IPL, inner plexiform layer; INL, inner nuclear layer; OPL, outer plexiform layer; ONL, outer nuclear layer; P, postnatal day; WT, wild type; TRX-Tg, thioredoxin-1 transgenic. Each group, *n* = 5.

To examine retinal damage, we analyzed the histology of retinal sections stained with hematoxylin and eosin in P12, P17, and P28 mice. We assessed the number of cells in GCL and the thickness of IPL, INL, OPL, and ONL. We assessed the central part of the retina, which is close to the optic disc, and the peripheral part, which is 500 μm away (Figure 6A). From the previous research, the OIR model mouse demonstrates a thinner layer of IPL, INL, and OPL [30]. Another study of the ROP model mouse reveals a thinner layer in INL and ONL [28]. In our study, we compared the thickness of each layer with the ratio of total layer thickness divided by each layer because we aimed to avoid the different thicknesses by the cutting angle. According to the previous studies, only the GCL layer was examined by counting the number of cells [28,30]. On P12 and P28, the WT retina subjected to hyperoxia showed a reduction in the thickness of the central region of the INL compared to room air (0.30 (±0.03) vs. 0.35 (±0.03), *p* < 0.05; 0.23 (±0.02) vs. 0.28 (±0.03), *p* < 0.05, respectively). However, the TRX-Tg mice did not show any differences between the retina exposed to hyperoxia and room air on P12 and P28 (0.35 (±0.03) vs. 0.34 (±0.03), *p* = 0.69; 0.24 (±0.03) vs. 0.26 (±0.03), *p* = 0.54). The ONL layer of the retina in WT mice grew in thickness in the central region of the retina under hyperoxic circumstances on P28 compared to room air conditions (0.47 (±0.03) vs. 0.39 (±0.02), *p* < 0.01). However, the retinas exposed to hyperoxia and room air in TRX-Tg mice did not show a significant difference (0.44 (±0.02) vs. 0.40 (±0.03), *p* = 0.05) (Figure 6B). In contrast, there were no differences in the peripheral part of the retina between each group (Figure 6C).

## 4. Discussion

Hyperoxia was used in this study to develop OIR mice, which are ROP mouse models. OIR mice demonstrated increased avascular area, neovascular area, morphological abnormalities of arteries and veins, retinal vascular permeability, and thinning of retinal tissue by controlling gene expressions of proinflammatory and angiogenic factors in the retina. These abnormalities in OIR mice were reduced by TRX-Tg. In the OIR mouse model, the mechanism of retinal vascular growth is critical for pathogenesis. With 75% oxygen supply, the retinal microvasculature begins to degenerate (vaso-obliteration phase). After return to room air environment, the retinal tissue becomes relatively hypoxic, and ischemia predisposes to abnormal compensatory neovascularization starting at the succeeding phase (neovascularization phase).

P12 was set to examine the role of hyperoxia on the mouse retina. The TRX-Tg mouse retina subjected to hyperoxia during the vaso-obliteration phase had a much smaller avascular area than the WT mouse retina exposed to hyperoxia, according to this study. This reduction in avascular area in the TRX-Tg mouse retina after hyperoxic exposure persisted during the succeeding phase, owing to the fact that the avascular area was smaller in TRX-Tg than in WT mice from the beginning. We also discovered that, in the central part of the retinal membrane, the WT mice exposed to hyperoxia revealed a significantly thinner layer than those exposed to normoxia. The disparities in the thickness of the retinal membrane layer between mice subjected to hyperoxia and mice exposed to room air vanished in TRX-Tg mice. This is because the INL has a particularly large number of blood vessels and the thickness of the layer is considered proportional to the blood vessel supply [31]. We believe that overexpression of TRX protects retinal development based on these findings. Moreover, the gene expressions of VEGF-A, IGF-1, and CXCL2, which are associated with neovascularization, were significantly reduced after hyperoxic exposure in WT mouse retinas. In TRX-Tg mice retinas exposed to hyperoxia; however, there was no significant reduction in VEGF-A and CXCL2 gene expression. VEGF-A is a hypoxia-inducible growth factor that is inhibited in hyperoxic environments [32,33,34]. This finding suggests that the TRX-Tg mouse retina can prevent the decrease in VEGF-A levels due to hyperoxia exposure. Several studies have suggested that, in different mammalian cell lines, TRX overexpression increases not the activity but also the stability of hypoxia-inducible factor-1alpha, a transcription factor located upstream of VEGF-A [35,36], implying that TRX overexpression may regulate the expression levels of VEGF-A via the stability of hypoxia-inducible factor-1alpha. CXCL2 is a cytokine that is related to the chemical attraction of neutrophils, involved in many immune responses, and also induced expression during angiogenesis [37]. From the results of these morphological and gene expression analyses, overexpressed TRX prevents the suppression of gene expression of angiogenic factors, such as VEGF-A and CXCL2, according to the findings of these morphological and gene expression investigations. This could have aided in the prevention of avascular region formation, resulting in less retinal ischemia in TRX-Tg mice after hyperoxic exposure. Increased IL-6 expression levels in the retina exposed to hyperoxia have been reported in previous investigations [38]. Anti-inflammatory activity is also recognized to be significant in this pathological condition. In this study, WT mice exposed to hyperoxia increased IL-6 expression levels during the vaso-obliteration phase. However, in the TRX-Tg mouse, there was no change in the IL-6 expression levels between retinas exposed to hyperoxia and room air, suggesting that during the vaso-obliteration phase, TRX helps to reduce retinal inflammation. This anti-inflammatory effect of TRX overexpression may be explained in part by the direct interaction of TRX with macrophage migration inhibitory factor, which is known to regulate the expression of proinflammatory mediators [39].

When compared to the WT retina, the TRX-Tg retina had a smaller avascular region and less neovascular formation after hyperoxia. Increased aberrant neovascularization was seen in the OIR mouse model in studies using the neovascular region [29]. This study shows that the TRX-Tg mouse retina reduces the increase in the avascular area caused by hyperoxic exposure and neovascular area during recovery demonstrating the atypical creation of vessels during the neovascularization phase. Hyperoxia exposure significantly reduced the number of veins in the WT mouse retina, whereas no significant difference between the TRX-Tg mouse retina exposed to hyperoxia and room air was noted. Moreover, from the histological viewpoint, the TRX-Tg reveals a protective effect on thinning the INL in the retina induced by hyperoxia exposure, and the retina demonstrated thickened ONL under the hyperoxic conditions at P28 in the central region. The thickness of INL and ONL in TRX-Tg mice’s retinas exposed to hyperoxia and room air did not change. Since the choroidal vascular bed primarily supplies the ONL in the retina, the thickness of the ONL may be related to the vascular development of the choroidal vascular bed [40]. Unfortunately, our study did not assess the choroidal vasculature. As a result, although this mechanism cannot be fully explained, TRX-Tg can mitigate the effects of hyperoxia exposure on pathological changes in retinal neovascularization. During the neovascularization phase, VEGF-A expression levels increased in WT mouse retinas after hyperoxia exposure, but there was no change in VEGF-A expression in TRX-Tg mice retinas exposed to hyperoxia, suggesting that overexpressed TRX might block retinal neovascularization. Analysis of the HO-1-Tg mouse lungs suggests a mild increase in HO-1 has cytoprotective effects [41]. In this report, HO-1 gene expression levels were slightly increased in the TRX mouse retina than in the WT mouse retina during the neovascularization phase after hyperoxia exposure. The mild increase in HO-1 expression in HO-1 expression in the overexpressed TRX-Tg mouse retina suggests that it may protect retinal cells during the neovascularization phase. Furtheremore, hyperoxic exposure reduced gene expression levels of IGF-1 and Angpt1 in the WT mouse retina but not in the TRX-Tg mouse retina. IGF-1 modulates vascular survival and endothelial growth, and Angpt1 plays a crucial role in forming the retinal blood vessel network during postnatal development [37]. These findings suggest that overexpressed TRX not only suppresses the expansion of the avascular field during the vaso-obliteration phase but also suppresses the formation of abnormal retinal neovascularization during the neovascularization phase by avoiding changes in gene expression of angiogenic factors and regulating the vascular system requires for retinal development.

One of the other important pathogeneses of ROP is vascular leakage. Previous studies using the OIR mouse model have reportedly increased blood leakage from retinal blood vessels and increased expression of tight junction proteins [30,42]. The retinal vascular permeability is influenced by the blood–retinal barrier, which is made up of vascular endothelial cells. The barrier properties of the vessel wall are produced by tight junction proteins, such as claudin-5 and occludin, between vascular endothelial cells [30,43]. The retinal vascular permeability was assessed using Hoechst 33342 and FITC-dextran during the recovery phase of P28. From morphological analysis, TRX-Tg retina had less extravasation of Hoechst 33342 stain than WT mice after hyperoxia exposure. In addition to vascular staining, we carried out a western blot analysis of tight junction protein on P28. Previous studies have reported overexpression of claudin-5 and occludin in the OIR model at week 8 [30]. However, in our study, contrary to the hypothesis that TRX-Tg preserves vascular permeability by modulating the expression of tight junction proteins after hyperoxia exposure, the expression levels of tight junction proteins did not reveal a significant difference among any groups. We assumed that there was a possibility due to the difference in the timing of the evaluation. The mechanism of enhanced vascular permeability of retinal blood vessels due to hyperoxia exposure and repression by overexpressed TRX-Tg will need to be investigated further in the future.

## 5. Conclusions

During the vaso-obliteration phase, TRX-Tg reduced the probability of avascular area enlargement owing to hyperoxic exposure and prevented avascular area expansion, reduced neovascularization, and increased vascular permeability during the neovascularization phase. The beneficial effects of overexpressed TRX on these OIRs may result from the regulation of gene expression of pro-inflammatory cytokines and angiogenic factors. As a result, TRX could be used in the future as a preventive or therapeutic treatment for ROP.

## Data Availability

The data presented in this study are available in the article.
